# Low dose NSAIDs and sysadoas in the management of knee osteoarthritis

**DOI:** 10.1007/s40520-025-03221-2

**Published:** 2025-11-06

**Authors:** Alberto Migliore, Orazio De Lucia, Alessandro de Sire, Andrea Šajbidor, Ladislav Šenolt, Sándor Szántó, Joan Calvet Fontova, Johannes Flechtenmacher, Ali Mobasheri, Jordi Monfort Faure, Jean-Yves Reginster, Nicola Veronese

**Affiliations:** 1 Rheumatology Unit, San Pietro Fatebenefratelli Hospital, Rome, Italy; 2 Department of Rheumatology and Medical Sciences, Clinical Rheumatology Unit, ASST Gaetano Pini-CTO, Milan, Italy; 3 Physical and Rehabilitative Medicine, Department of Medical and Surgical Sciences, University of Catanzaro “Magna Graecia”, Catanzaro, 88100 Italy; 4 Research Center on Musculoskeletal Health, MusculoSkeletalHealth@UMG, University of Catanzaro “Magna Graecia”, Catanzaro, 88100 Italy; 5 Špecializovaná Nemocnica Pre Ortopedickú Protetiku, Záhradnícka 42, Bratislava, 821 08 Slovakia; 6 Orthopedic Clinic, Bratislava, Slovakia; 7 Institute of Rheumatology, Na Slupi 4, Prague, 128 00 Czech Republic; 8 Department of Rheumatology, First Faculty of Medicine, Charles University, Prague, Czech Republic; 9 Faculty of Medicine, Department of Sports Medicine, University of Debrecen, Debrecen, Hungary; 10 Department of Rheumatology, Medicine Department, Parc Taulí Hospital Universitari, Institut d’Investigació i Innovació Parc Taulí (I3PT-CERCA), Universitat Autònoma de Barcelona, Sabadell, Spain; 11 Ortho-Zentrum Karlsruhe, Waldstrasse 67, 76133 Karlsruhe, Germany; 12 Research Unit of Health Sciences and Technology, Physics and Technology, University of Oulu, Oulu, Finland; 13 Department of Regenerative Medicine, State Research Institute Centre for Innovative Medicine, Vilnius, Lithuania; 14 Department of Joint Surgery, Sun Yat-sen University, Guangzhou, People’s Republic of China; 15 Department of Rheumatology, Institut Hospital del Mar d’Investigacions Mèdiques, Passeig Marítim de la Barceloneta, 25, 29, 08003 Barcelona, Spain; 16 Protein Research Chair, Biochemistry Department, College of Science, King Saud University, Riyadh, Kingdom of Saudi Arabia; 17 Saint Camillus International University of Health Sciences, Rome, Italy; 18 Unità Locale Socio Sanitaria 3 Serenissima, Primary Care Department, Dolo Mirano, Venice, Italy

**Keywords:** Osteoarthritis, Knee osteoarthritis, Diclofenac, Chondroitin sulfate, Low dose diclofenac, NSAIDs

## Abstract

**Introduction:**

Osteoarthritis (OA) is a chronic, progressive joint disease characterized by the degradation of articular cartilage, subchondral bone remodeling, synovial inflammation, and osteophyte formation. Despite being a leading cause of disability in older people, effective long-term management of OA remains a significant challenge. Current treatment strategies primarily focus on symptom control, with both non-pharmacological and pharmacological interventions.

**Materials and methods:**

A systematic literature review followed by a structured Delphi survey was conducted, involving an international Technical Expert Panel (TEP) of OA specialists. The panel evaluated the efficacy, safety, and clinical utility of combining low dose diclofenac and chondroitin sulfate in OA management.

**Results:**

The analysis of expert consensus indicated that the combination of low dose diclofenac and chondroitin sulfate may be effective in reducing pain and improving joint function in patients with knee OA. Additionally, this combination could reduce the need for higher doses of NSAIDs, thereby minimizing systemic adverse effects.

**Conclusion:**

The combination of low dose diclofenac and chondroitin sulfate represents a promising therapeutic strategy for managing knee OA. Further studies are needed to confirm these findings and optimize therapeutic strategies to improve patient outcomes.

**Supplementary Information:**

The online version contains supplementary material available at 10.1007/s40520-025-03221-2.

## Introduction

Osteoarthritis (OA) is a chronic, progressive, and multifactorial joint disease that represents one of the most prevalent musculoskeletal disorders worldwide [1, 2]. It is characterized by the degradation of articular cartilage, subchondral bone remodeling, synovial inflammation, and osteophyte formation, leading to pain, stiffness, and functional impairment. OA primarily affects weight-bearing joints, such as the knees and hips, but can also involve other joints, significantly impacting mobility and quality of life [3, 4]. With the global increase in life expectancy and rising obesity rates, the prevalence of OA continues to grow, placing a substantial burden on healthcare systems and social care infrastructures [1].

Despite being a leading cause of disability in older people, effective long-term management of OA remains a major challenge [3]. Current treatment strategies primarily focus on symptom control, aiming to alleviate pain, improve joint function, and slow disease progression. Non-pharmacological interventions, such as physical therapy and weight management [5], are widely recommended as first-line approaches [3, 6]. However, pharmacological treatments, including analgesics, nonsteroidal anti-inflammatory drugs (NSAIDs), and chondroprotective agents, play a crucial role in managing symptoms and preserving the integrity of articular cartilage, particularly in moderate-to-severe OA cases [7].

Among the available pharmacological options, chondroitin sulfate, a natural component of cartilage extracellular matrix, has been extensively studied for its potential disease-modifying and symptom-relieving properties. High quality formulation of SYSADOAs (symptomatic slow-acting drugs for osteoarthritis) at correct dosages has demonstrated chondroprotective and mild anti-inflammatory effects, with evidence suggesting its ability to reduce cartilage degradation and improve joint function [8]. On the other hand, **low dose** diclofenac has been used to give anti-inflammatory action while minimizing systemic adverse effects, making it a valuable option for patients requiring long term use [9].

Although both SYSADOAs and different dosages of diclofenac have individually shown efficacy in OA management, limited evidence exists regarding the potential benefits of their combined use [10]. Given the complementary mechanisms of action—chondroitin sulfate targeting cartilage preservation and diclofenac providing rapid pain relief—there is a strong rationale for evaluating their synergistic effects. However, consensus on their combined efficacy and safety remains undefined, highlighting the need for expert-driven evaluations to guide clinical decision-making.

To address this knowledge gap, a systematic literature review followed by a structured **Delphi survey** was conducted, involving an international Technical Expert Panel (TEP) of OA specialists. The **Delphi method** is a widely used approach for achieving expert consensus through an iterative process of structured questionnaires and controlled feedback [11]. This method is particularly valuable for addressing controversial or emerging topics in medicine where clinical evidence is still evolving. The survey applied the **RAND/UCLA Appropriateness Method**, a validated framework for assessing expert agreement [12], to systematically evaluate the role of the combination in OA treatment.

The primary aim of this TEP was to determine the level of agreement among experts regarding the efficacy, safety, and clinical utility of combining **low dose** diclofenac and chondroitin sulfate in OA management. By analyzing expert consensus, this research seeks to provide clinically relevant insights that may contribute to optimize therapeutic strategies and improve patient outcomes. The findings of this Delphi survey could serve as a foundation for further studies and recommendations in the field of OA treatment.

## Materials and methods

The TEP, composed of 5 rheumatologists, 2 orthopedics, 1 specialist in sport medicine, 1 physiatrist, 1 musculoskeletal physiologist and 1 geriatrician, paid great attention to achieve consensus through rigorous scientific methodology.

As part of the Delphi survey, a Systematic Literature Review (SLR) was conducted by S.M.P. (Science Librarian) to examine the use of Condrosulf and Flectorgo in patients with osteoarthritis (OA) and to identify predictors of both positive and negative clinical response.

The steering committee of the TEP including AM (Scientific Coordinator & Steering Committee), ODL, LS, AS, SS and AdS identified the clinical questions for the SLR, formulating them according to the Patient, Problem or Population/Intervention/Comparison, control or comparator/Outcome(s) (PICO) [Timing, duration or date of publication (T)/Study type (S)] format. Table 1 presents the structure of the PICOS research model applied in the review.

**Table 1:** Formulation of clinical questions in PICOS format

PICOS	Human Patients, Adults, Osteoarthritis
I	Oral treatment, diclofenac, NSAID, chondroprotection, Chondroitin sulfate, SYSADOA, combined treatment
C	–
O	All
S	Reviews, Narrative Reviews, Meta-Analysis (11); Randomized Control Trials (6); Prospective studies (2); Retrospective studies (1); Cohort studies (1)

**Fig. 1:**
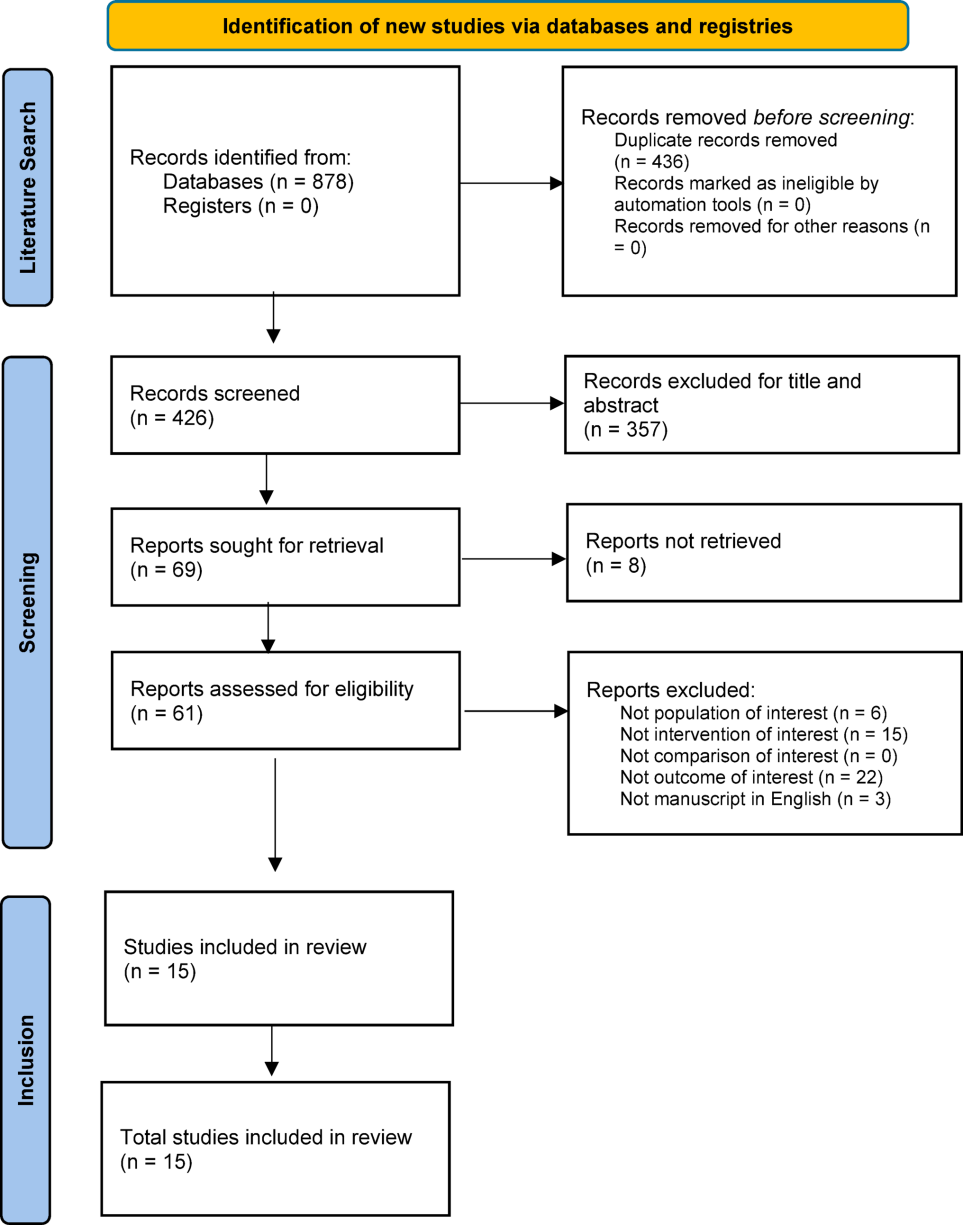
Selection of paper during the SLR

To ensure a comprehensive and unbiased selection of literature, the following databases were interrogated: PubMed/Medline, Embase, Cochrane Library. The research exclusively included studies on adult human subjects, without language or publication date restrictions, minimizing the risk of bias due to these factors (Fig. 1). An ad hoc extraction module was developed to systematically collect relevant data from each study. The extraction module captured key study characteristics, including: author, year, type of study, disease and site, type of SYSADOA/NSAID used, number of participants, endpoints, follow-up period, safety and observations [see tables in supplementary material available online].

The retrieved studies were carefully reviewed by the panellists, who selected a subgroup of studies deemed particularly relevant for further assessment. Both quantitative and qualitative syntheses of the extracted data were conducted. The characteristics of the populations enrolled in the selected studies were analyzed.

To ensure the accuracy and reliability of the review process, two independent experts conducted separate evaluations of each study. In cases of discrepancies, a third expert was consulted to reach a final decision. The quality of the selected studies was assessed using the 2018 version of the Mixed Methods Appraisal Tool (MMAT), evaluating key methodological aspects such as study aim appropriateness, research methodology, study design, data collection and analysis methods, and the clarity of reported findings.

The retrieved papers were analyzed and evaluated using a structured data extraction form. The steering committee of the TEP then selected 19 key statements, which were discussed in a first plenary meeting held on web (July 2024).

During a following meeting, the TEP refined the statements, ultimately defining 31 statements, which were further assessed through two rounds of a Delphi survey. The results, including any areas of disagreement, were reviewed and discussed in a final plenary web session in December 2024, where a final consensus was reached.

## Results

Considering the results of the SLR, the steering committee proposed 31 statements divided into six domains. During the first TEP meeting, the scientific evidence retrieved was presented and discussed. At the end of the meeting, the TEP defined a questionnaire composed of 33 statements grouped into six domains.

The questionnaire was discussed in two rounds of Delphi survey to find an agreement among the participants. The agreement with each statement was defined according to the RAND/UCLA criteria, with a vote from 1 (total disagreement) to 9 (total agreement). The agreement and disagreement among the experts were defined as follows: agreement if 80% of the 14 panellists’ ratings were concentrated in one of the 3-point regions (1–3; 4– 6; 7–9); disagreement if 90% of the 14 panellists’ ratings were spread across one of the two extra-wide regions (1–6 or 4–9).

The whole process was carried out using a web platform. The results of the questionnaire were discussed by the TEP during the second plenary meeting. Unanimity was reached in the domains, and 33 items (Table 2) were selected.

**Table 2:** Domains and items approved after discussion

Domain	Item	Level of aggregation (%)		
**1**	**Area 1 - Overarching considerations (principles)**	**% 7–9**	**% 4–6**	**% 1–3**
1.1	Osteoarthritis (OA) is a multi-form disease with many phenotypes	100,0%	0,0%	0,0%
1.2	In osteoarthritis, a low level of synovial inflammation is present in variable degrees	90,0%	10,0%	0,0%
1.3	In addition to the short-term goals of treating OA (e.g. pain relief and improving musculoskeletal function), it is important to slow down structural damage	90,9%	9,1%	0,0%
1.4	The treatment of OA requires long-lasting and therefore safe therapies	100,0%	0,0%	0,0%
**2**	**Area 2 - Rationale of NSAIDs/SYSADOAs combination in knee osteoarthritis**	**% 7–9**	**% 4–6**	**% 1–3**
2.1	NSAIDs are recommended in all guidelines of both international and national societies	90,9%	9,1%	0,0%
2.2	SYSADOAs are recommended in some guidelines of both international and national societies	90,9%	0,0%	9,1%
2.3	The use of SYSADOAs in patients taking systemic NSAIDs is also recommended in order to reduce the consumption of NSAIDs	90,9%	0,0%	9,1%
2.4	Studies on NSAIDs suggest they could not be considered a homogeneous class	100,0%	0,0%	0,0%
2.5	A treatment model including SYSADOAs, NSAIDs, exercise and rehabilitative interventions can be useful to improve joint function	100,0%	0,0%	0,0%
**3**	**Area 3 - Efficacy and safety of low-dose NSAIDs**	**% 7–9**	**% 4–6**	**% 1–3**
3.1a	Use of low-dose NSAIDs may be a feasible option for the flare-ups treatment of mild to moderate knee osteoarthritis	90,0%	10,0%	0,0%
3.1b	Use of low-dose NSAIDs may be a feasible option for the prolonged treatment of mild to moderate knee osteoarthritis according to patient’s specific factors	90,0%	10,0%	0,0%
3.2	A low-dose regimen of diclofenac ranging from 12.5 to 25 mg per day has been shown to be effective in some clinical trials on musculoskeletal pain	90,0%	10,0%	0,0%
3.3	Common daily doses in clinical practice of oral diclofenac range from 75 to 150 mg	100,0%	0,0%	0,0%
3.6	The safety of low-dosing regimen of diclofenac to control symptoms in osteoarthritis should be considered on an individual patient basis	90,9%	9,1%	0,0%
3.7	Mucosal injury with diclofenac-K liquid 25 mg liquid capsules is significantly lower than ASA 500 mg/day	90,9%	9,1%	0,0%
**4**	**Area 4 - Efficacy and safety of SYSADOAs**	**% 7–9**	**% 4–6**	**% 1–3**
4.1	Oral supplementation with pharmaceutical-grade glucosamine or chondroitin sulfate reduces pain in knee OA	81,8%	18,2%	0,0%
4.2	SYSADOAs systemic slow acting drugs for osteoarthritis (chondroitin sulfate, glucosamine, etc.) can be considered safe treatments for patients with knee OA	81,8%	9,1%	9,1%
4.3	Oral supplementation with glucosamine or chondroitin sulfate may improve function in knee OA	81,8%	18,2%	0,0%
4.4	There is clinical evidence suggesting that pharmaceutical grade products of some SYSADOAs (chondroitin and glucosamine) can slow the radiological progression of OA	90,0%	0,0%	10,0%
4.5	Chondroitin sulfate is a potential first-line treatment for the management of patients with mild to moderate OA in knee and/or in other sites	81,8%	9,1%	9,1%
4.6	Efficacy of 800 mg chondroitin sulfate is similar to 200 mg celecoxib in reducing pain and improving function in knee OA patients with radiological Kellgren and Lawrence grade 1–3	90,9%	0,0%	9,1%
**5**	**Area 5 - Efficacy and safety of chondroitin sulfate combined with NSAID in knee OA**	**% 7–9**	**% 4–6**	**% 1–3**
5.1	The concomitant use of chondroitin sulfate with NSAID is suggested in order to improve clinical efficacy in patients with knee OA	81,8%	18,2%	0,0%
5.2	Both crystalline glucosamine sulfate and chondroitin sulfate decrease the use of NSAIDs in pharmaco-epidemiology studies in patients with knee OA	81,8%	18,2%	0,0%
5.3	The concomitant use of chondroitin sulfate with a NSAID may be cost-effective because the combination reduces the cumulative dose of NSAIDs and related adverse events	90,9%	9,1%	0,0%
**6**	**Area 6 - Efficacy and safety of chondroitin sulfate combined with low-dose diclofenac in knee OA**	**% 7–9**	**% 4–6**	**% 1–3**
6.1	Use of Chondroitin Sulfate combined with low-dose diclofenac may contribute to reduce pain in knee OA	90,9%	9,1%	0,0%
6.2	Use of Chondroitin Sulfate combined with low-dose diclofenac allows therapy to be personalized by modulating the NSAID dose according to the patient’s inflammatory status and Kellgren-Lawrence grade	90,9%	9,1%	0,0%

### Overarching considerations

All panellists agree that OA is a multifaceted disease with different phenotypes, as emerging from recent literature [13, 14], and that it requires treatment aimed both at alleviating incident pain and slowing down structural damage [3]. To achieve the latter goal, continuous long-term therapies are necessary, which, due to their prolonged use, must have a high safety profile. This statement is particularly relevant considering that OA patients are often affected by multi-morbidity, taking multiple medications, and are of advanced age. Surprisingly, in the first round, there was no strong agreement on the notion that OA is a low-grade inflammatory disease, despite substantial literature spanning the past two decades on the topic [15]. Agreement was reached when the statement was simplified by removing references to disease stages and phenotypes, emphasizing that the inflammatory phenomenon is present across all phenotypes and disease stages.

### Rationale of NSAIDs/SYSADOAs combination in knee osteoarthritis

Regarding the rationale for the use of anti-inflammatory drugs and SYSADOAs in knee OA, there is unanimous consensus in considering anti-inflammatory drugs as essential for the OA management. This is clearly reflected in national and international guidelines on knee OA treatment ( [16–22]).

While direct comparisons are limited, the overall evidence base for SYSADOAs is considered less robust than that for NSAIDs. Consequently, their use is endorsed mainly by specific international bodies, such as the ESCEO [20], while others, such as NICE [23], OARSI [19], and ACR [18], discourage their use. Additionally, some organizations remain uncertain (EULAR [24], SIOT [16], AAOS [21]). However, SYSADOAs have demonstrated an undefined degree of anti-inflammatory, and chondroprotective activity [8, 25, 26] and an optimal safety [27]. Based on this, one of the rationales for using SYSADOAs is to reduce the need for anti-inflammatory drugs [10].

A debated statement concerns whether NSAID constitute a homogeneous class. The initial draft included the phrase “a non-homogeneous class with different relative risks,” based on the review by Magni et al. [22]. This statement reached consensus only after the second Delphi round, following the removal of the final part regarding different relative risks and maintaining “non homogeneous class”.

The last and most comprehensive statement, which immediately reached unanimous agreement, is that the osteoarthritis treatment model includes not only pharmacological therapy, but also physical exercise and rehabilitative interventions aimed at improving joint function. This reflects the multimodal approach endorsed by all scientific societies [19, 20, 23].

### Efficacy and safety of low-dose NSAIDs

Regarding the use of low-dose NSAID for disease flares and for prolonged periods in the treatment of osteoarthritic pain, these topics were subject to discussion and were only accepted at the end of the second round of the Delphi process. The combination of disease flares with prolonged treatments did not convince the voters, leading to the separation of the two statements.

During the meetings, attention was focused on two key aspects. First, the goal of controlling pain associated with disease flares using the lowest possible dose of anti-inflammatory drugs [9, 28, 29], a concept endorsed by all international [18, 19] and national [16, 30] guidelines. Second, there was a shared consensus that low-dose anti-inflammatory drugs may be an option for more prolonged periods, but only after an individualized patient assessment. This approach considers the extensive literature on the gastrointestinal and cardiovascular risks associated with long-term use of NSAIDs [31, 32], which can be mitigated by reducing both the dosage of the anti-inflammatory agent [33] and the duration of exposure to the drug.

In line with the objective of this study, further clarification was provided: low-dose diclofenac is defined as a daily dose ranging from 12.5 to 25 mg, whereas the standard dosage of diclofenac ranges from 75 to 150 mg per day.

Finally, it was emphasized that, although low-dose diclofenac represents a therapeutic option with a favorable safety profile compared to other NSAIDs [9, 34], the final decision on prescription should always consider the individual patient’s comorbidities.

### Efficacy and safety of sysadoas

There is broad, though not unanimous, consensus on the analgesic activity exerted by glucosamine and chondroitin sulfate when administered at a pharmacological dosage [4, 22, 26, 35]. In an initial version that did not specify the pharmacological dosage, several members of the steering committee advocated for its explicit inclusion to distinguish it from a large number of over-the-counter products that do not clearly specify the dosages of their active ingredients [36].

Chondroitin sulfate and glucosamine are considered treatments with a high safety profile, and their oral supplementation may improve knee function in osteoarthritis [37, 38]. There is also evidence suggesting that these compounds may slow the radiographic progression of osteoarthritis [39, 40].

Given these premises, pharmacological-grade chondroitin sulfate, the focus of our research, may represent a first-line treatment option for knee pain in mild to moderate osteoarthritis [40]. Furthermore, there is evidence suggesting that a daily dose of 800 mg of chondroitin sulfate may be comparable to 200 mg of celecoxib in reducing pain associated with mild to moderate knee osteoarthritis [41, 42].

### Efficacy and safety of chondroitin sulfate combined with NSAID in knee osteoarthritis

Combining chondroitin sulfate with nonsteroidal anti-inflammatory drugs (NSAIDs) has been explored to enhance clinical efficacy in patients with knee osteoarthritis (OA). Studies suggest that this combination may offer benefits in pain reduction and functional improvement reducing NSAIDs intake [43].

A meta-analysis published in the Archives of Internal Medicine reported that the combination of glucosamine or chondroitin with NSAIDs at lower cumulative doses than when used alone shows better efficacy in reducing pain associated with OA [44].

The concomitant use of chondroitin sulfate with NSAIDs may be beneficial both clinically and economically for patients with knee OA. This combination could reduce the cumulative dose of NSAIDs required, thereby decreasing the risk of gastrointestinal adverse events associated with NSAID use [10]. Additionally, a cost-effectiveness analysis showed that the use of chondroitin sulfate not only improves clinical symptoms but is also associated with a reduction of overall care costs, primarily due to decreased NSAIDs use and related side effects [10].

### Efficacy and safety of chondroitin sulfate combined with low-dose diclofenac in knee osteoarthritis

Given the premises outlined in the previous paragraphs, there has been unanimous consensus from the beginning in considering the combination of a low dose of diclofenac and chondroitin sulfate as beneficial in reducing pain in patients with knee OA [10] despite the lack of literature on this specific topic. In addition to its efficacy, it has also been emphasized that this combination allows for a personalized approach, enabling fine-tuned modulation of the anti-inflammatory dosage administered to each individual patient [45].

## Discussion

The Delphi consensus process has confirmed that a combined treatment approach utilizing low-dose NSAIDs and SYSADOAs, particularly chondroitin sulfate, can be both effective and safe for managing knee osteoarthritis. This approach offers the advantage of personalized treatment, allowing clinicians to adjust the anti-inflammatory dosage according to each patient’s specific needs. Personalization is crucial in the treatment of OA, as patients may respond differently to medications and exhibit varying tolerances to side effects. This flexibility enables clinicians to optimize treatment efficacy while minimizing the risk of adverse effects. Moreover, combining low-dose NSAIDs with SYSADOAs reduces the need for higher NSAID doses, which are often linked to a higher incidence of adverse events. This is particularly important for OA patients, who often require long-term management of their condition. The combined strategy of low-dose NSAIDs and SYSADOAs may provide an effective treatment option for knee OA, allowing a cumulative effect of the therapeutic target of each one: the control of low-degree inflammation and the protective effect on cartilage. However, further evidence is required to substantiate this hypothesis. Careful evaluation of each patient’s characteristics is essential when initiating therapy to avoid the risks associated with long-term NSAID use in the perspective of the inflammation control along the evolution of the disease. This underscores the importance of personalized medicine in OA management, taking into account each patient’s unique needs and potential risks. By integrating both pharmacological treatments with two different products having different mechanism of action and biological target and non-pharmacological treatments, clinicians can optimize patient outcomes while minimizing the risks associated with high dosage of NSAIDs. This whole approach to osteoarthritis management highlights the value of combining multiple treatment modalities to enhance patient outcomes.

The use of SYSADOAs in the treatment of OA remains a subject of ongoing debate within the scientific community. Several issues are currently under discussion. Some efficacy concerns are reported. Some studies suggest that prescription-grade SYSADOAs, such as glucosamine and chondroitin sulfate, may provide pain relief and improve joint function in OA patients. However, the evidence is inconsistent, if you consider RCTs or Meta-analysis involving non-prescription-grade SYSADOAs, these studies report minimal or no benefits compared to placebo [46–48]. There is a wide variability in study results, due to differences in study design, patient populations, and the specific formulations of SYSADOAs. This variability complicates the ability to draw definitive conclusions regarding their effectiveness [49]. Major medical societies, including the American College of Rheumatology (ACR) [18] and the Osteoarthritis Research Society International (OARSI) [19], offer varying guidelines on the use of SYSADOAs. Some recommend their use in certain patient subgroups, while others caution against routine use due to a lack of robust evidence. Other Societies or recommendations like ESCEO [20], EULAR [24], SIOT [16], AAOS [21] recommend SYSADOAs use in the complex OA management. However, it is well recognized that SYSADOAs are generally considered safe, with a low incidence of side effects, which is a favorable aspect, particularly for long-term use in chronic conditions such as OA [27].

Further research is essential to gain a clearer understanding of the potential benefits of SYSADOAs, identify patient subgroups who may benefit most, and determine the optimal dosing and duration of treatment. In summary, while SYSADOAs hold promise for some OA patients, the scientific community agrees that additional high-quality research is needed to solidify their role in clinical treatment guidelines.

However, in this work some limitations must also be acknowledged. The evidence base for the combined use of low-dose diclofenac and chondroitin sulfate remains scarce, as most clinical studies have investigated these agents separately rather than in combination. As a result, the consensus statements are grounded primarily in indirect evidence and expert opinion, rather than robust randomized controlled trials which are necessary to provide valid scientific evidence. It should also be noted that this consensus was developed by European experts, and recommendations might differ in other regions of the world depending on the availability of active compounds on local markets. In addition, heterogeneity in study designs, patient populations, and SYSADOA formulations across the literature complicates the interpretation and generalizability of findings. Although the Delphi methodology provides a structured and validated process for capturing expert consensus, it is inherently influenced by the panel’s composition and prior clinical experience. Finally, the lack of long-term outcome data and comparative effectiveness studies limits the ability to draw firm conclusions regarding durability of effect, safety in multimorbid patients, and cost-effectiveness. These limitations highlight the need for well-designed, adequately powered clinical trials to substantiate the preliminary consensus reached in this study.

Nevertheless, the group of international experts consulted on this issue has provided a clear and unanimous recommendation, suggesting it as a potential first-line treatment for mild-to-moderate knee osteoarthritis. Given the increasing average age of the population and the growing burden of comorbidities, this option, while not supported by robust scientific evidence, appears to have a rational basis for use.

## Supplementary Information

Below is the link to the electronic supplementary material.

Supplementary Material 1

Supplementary Material 2

Supplementary Material 3

Supplementary Material 4

## Data Availability

No datasets were generated or analysed during the current study.
