# Supplementary material for: Low dose NSAIDs and sysadoas in the management of knee osteoarthritis
Source: Aging Clin Exp Res. 2025 Nov 6;37(1):317. doi: 10.1007/s40520-025-03221-2 (PMC12592241; doi:10.1007/s40520-025-03221-2)
Supplement: Supplementary file 1 — Supplementary Material 1 [file 40520_2025_3221_MOESM1_ESM.docx]

| **Author/Year** | **Type of Study** | **Disease/Where** | **Sysadoa** | | **NSAID** | | **n patients** | **Endpoint 1** | **Endpoint 2** | **Follow up** | **Safety** | **Observations** |
| --- | --- | --- | --- | --- | --- | --- | --- | --- | --- | --- | --- | --- |
|  |  |  | **Active ingredients** | **Dosage** | **Drug** | **Dosage** |  |  |  |  |  |  |
| **1** Rubio-Terres 2010 | retrospective study  VECTRA | OA | 233 patients received CS, 234 received NSAIDs and the remaining 63 patients took CS plus NSAIDs together for the treatment of osteoarthritis |  |  |  | 530 | the average cost per patient with osteoarthritis treated with chondroitin sulfate compared with NSAIDs for 6 months | the possible impact that the reduction NSAID use due to monotherapy with or combined administration of chondroitin sulfate treatment may have on the budget of the Spanish National Health System | 6-month period | 2,666 cases of gastrointestinal adverse events (including 90 serious adverse events) will have been avoided for every 10,000 patients treated with chondroitin sulfate instead of NSAID | The overall 6-month cost per patient given chondroitin sulfate was 141 € compared with 182 € when treated with NSAIDs. If during the forthcoming 3 years, 5%, 10%, and 15% of patients currently treated with NSAIDs would gradually be replaced by treatment with chondroitin sulfate, the expected savings for the Spanish National Health System during these 3 years would be over 38,700,000 €. chondroitin sulfate is a treatment for osteoarthritis with a lesser cost and better gastrointestinal tolerability compared with NSAIDs |
|  |  |  |  |  |  |  |  |  |  |  |  |  |
|  |  |  |  |  |  |  |  |  |  |  |  |  |
|  |  |  |  |  |  |  |  |  |  |  |  |  |
|  |  |  |  |  |  |  |  |  |  |  |  |  |
|  |  |  |  |  |  |  |  |  |  |  |  |  |
|  |  |  |  |  |  |  |  |  |  |  |  |  |
|  |  |  |  |  |  |  |  |  |  |  |  |  |
|  |  |  |  |  |  |  |  |  |  |  |  |  |
|  |  |  |  |  |  |  |  |  |  |  |  |  |
|  |  |  |  |  |  |  |  |  |  |  |  |  |
|  |  |  |  |  |  |  |  |  |  |  |  |  |
|  |  |  |  |  |  |  |  |  |  |  |  |  |
|  |  |  |  |  |  |  |  |  |  |  |  |  |
|  |  |  |  |  |  |  |  |  |  |  |  |  |
|  |  |  |  |  |  |  |  |  |  |  |  |  |
|  |  |  |  |  |  |  |  |  |  |  |  |  |
|  |  |  |  |  |  |  |  |  |  |  |  |  |
|  |  |  |  |  |  |  |  |  |  |  |  |  |
|  |  |  |  |  |  |  |  |  |  |  |  |  |
|  |  |  |  |  |  |  |  |  |  |  |  |  |
|  |  |  |  |  |  |  |  |  |  |  |  |  |
|  |  |  |  |  |  |  |  |  |  |  |  |  |
|  |  |  |  |  |  |  |  |  |  |  |  |  |
|  |  |  |  |  |  |  |  |  |  |  |  |  |
