# Supplementary material for: Low dose NSAIDs and sysadoas in the management of knee osteoarthritis
Source: Aging Clin Exp Res. 2025 Nov 6;37(1):317. doi: 10.1007/s40520-025-03221-2 (PMC12592241; doi:10.1007/s40520-025-03221-2)
Supplement: Supplementary file 2 — Supplementary Material 2 [file 40520_2025_3221_MOESM2_ESM.docx]

| **Author/Year** | **Type of Study** | **Disease/Where** | **Sysadoa** | | **NSAID** | | **N of patients** | **Endpoint 1** | **Endpoint 2** | **Follow up** | **Safety** | **Observations** |
| --- | --- | --- | --- | --- | --- | --- | --- | --- | --- | --- | --- | --- |
|  |  |  | **Farmaco** | **Dosaggio** | **Farmaco** | **Dosaggio** |  |  |  |  |  |  |
| **1** Trevisan 2023 | Prospective study | Muskoloskeletal pain |  |  | Diclofenac epolamine | 12.5-mg soft capsule formulation | 182 | to determine how effective subjects rated a new low dose diclofenac epolamine soft capsule formulation for treating their mild-to moderate acute musculoskeletal pain |  | In the day | Oral treatment with DHEP 12.5-mg soft capsules was well tolerated, with only two AEs reported, only one of which was potentially correlated with treatment (nausea of mild intensity, self-resolved within 20 min) | 90,7% of patients expressed overall satisfaction with the treatment, and the Investigators were satisfied with the treatment for 92.9% of subjects |
| **2** Hawkey 2012 | endoscopist-blinded, randomised, parallel-group study | Healthy subjects |  |  | Diclofenac K 25 vs diclofenac-K 12.5, acetylsalicylic acid and ibu profen | K 25 mg liquid capsules vs K 12.5 mg tablets, (ASA) 500 mg and 200 mg liquid capsules | 132 | To evaluate the gastro-duodenal tolerance of diclofenac-K 25 mg liquid capsules vs. diclofenac-K 12.5 mg tablets, acetylsalicylic acid (ASA) 500 mg tablets and ibu profen 200 mg liquid capsules |  | 15 doses of diclofenac-K 25 mg liquid capsules (n = 36), diclofenac-K 2 9 12.5 mg tablets (n = 36), ibuprofen 2 9 200 mg liquid capsules (n = 24) or ASA 2 9 500 mg tablets (n = 36) over 5 days | Adverse events were assessed throughout the study. In addition, haematology, blood chemistry and urine tests were conducted at screening |  |
| **3** Kienzler 2012 | randomized, open-label crossover, single-dose study | Healthy subjects |  |  | diclofenac-K | 12.5 liquid capsules or tablets | 42 | To compare the bioavailability of diclofenac-K 2 × 12.5 mg liquid capsules versus diclofenac K 2 × 12.5 mg tablets |  | 2 × 12.5 mg liquid capsules or tablets over two 1-day treatment periods separated by a 14-day washout period | Fewer than 5% of subjects experienced an AE after either treatment. No deaths, SAEs or other significant AEs were reported, no significant changes were observed in vital signs, and no emergent abnormalities were revealed dur ing physical examination | Diclofenac-K 12.5 mg liquid capsules and tablets were equivalent for overall sys temic exposure to diclofenac, as demon strated by the comparable AUC. However, diclofenac was absorbed more rapidly from the diclofenac-K 12.5 mg liquid capsule, with a Cmax that was almost double that of the diclofenac-K 12.5 mg tablet |
| **4** Zuniga 2011 | two multicenter, parallel group, double-blind, placebo-controlled studies | Patients undergoing third molar extraction and experiencing a requisite level of pain (≥50 mm on a 100-mm VAS 4h post-surgery) |  |  | Diclofenac potassium soft gelatin capsules (DPSGC) | randomized to receive single doses of DPSGC 25 mg, 50 mg, 100 mg, or placebo | 514 | Evaluate response of various doses of DPSGC in post-surgery dental patients |  | Single dose | DPSGC was well tolerated, and no serious adverse events were reported | Study design limitations include the short duration of the trial and evaluation of a relatively limited patient population |
| **5** Riff 2009 | Phase III, multicenter, random ized, double-blind, parallel-group, placebo-controlled study | Patients with first-metatarsal bunionectomy |  |  | DPSGC | 25 mg or placebo | 201 | The present study was conducted to as sess the efficacy and safety profile of DPSGC 25 mg in patients with pain after first-metatarsal bunionectomy |  | DPSGC 25 mg taken every 6 hours for 5 days | DPSGC was well tolerated, suggesting that it may be a practicable option for the treatment of mild to moderate acute pain | DPSGC 25 mg taken every 6 hours was effective in reducing postbunionectomy pain in the patients studied |
| **6** Moore 2007 | Review of 13 trials | treatment with OTC medication, for example, acute lower back pain, headache, acute pain after dental extraction, symptoms of cold and influenza (including fever), and dysmenorrhoea |  |  | Diclofenac | 12,5, 25 or 75 Liquid capsules or tablets | - | Evaluate different treatments of diclofenac in different situations and dosages |  | Various | incidence of adverse events in patients taking single or multiple doses of diclofenac potassium is similar to that of ibuprofen and placebo. In a safety study conducted to compare diclofenac potassium with ibuprofen for up to 3 months in patients with osteoarthritis of the knee, no differences in the pattern of adverse events were noted |  |
| **7** Dreiser 2003 | multiple dose, double-blind, double-dummy, randomized, placebo-controlled, parallel group trial | Back pain |  |  | Diclofenac K | 12.5 mg tablets vs ibuprofen and placebo | 124 (diclofenac) 122 (ibuprofen) 126 (placebo) | To assess efficacy and safety of diclofenac-K 12.5 mg tablets in the treatment of acute low back pain | Secondary outcomes for multiple dosing included time to rescue medication over the entire study, the End of Day global efficacy assessments (daily over Days 1-7), pain intensity differences on the VAS measured at Visit 2 and 3, and change in Eifel algofunctional index | The treatment consisted of an initial dose of 2 tablets followed by 1 or 2 tablets every 4-6 hours as needed (maximum 6 tablets/day) for 7 days |  | The flexible multiple dosing regimen of diclofenac-K 12.5 mg (initial dose of 2 tablets followed by 1-2 tablets every 4-6 hours, max. 75 mg/day) is an effective and safe treatment of acute low back pain |
|  |  |  |  |  |  |  |  |  |  |  |  |  |
|  |  |  |  |  |  |  |  |  |  |  |  |  |
|  |  |  |  |  |  |  |  |  |  |  |  |  |
|  |  |  |  |  |  |  |  |  |  |  |  |  |
|  |  |  |  |  |  |  |  |  |  |  |  |  |
|  |  |  |  |  |  |  |  |  |  |  |  |  |
|  |  |  |  |  |  |  |  |  |  |  |  |  |
|  |  |  |  |  |  |  |  |  |  |  |  |  |
|  |  |  |  |  |  |  |  |  |  |  |  |  |
|  |  |  |  |  |  |  |  |  |  |  |  |  |
|  |  |  |  |  |  |  |  |  |  |  |  |  |
|  |  |  |  |  |  |  |  |  |  |  |  |  |
|  |  |  |  |  |  |  |  |  |  |  |  |  |
