# Supplementary material for: Low dose NSAIDs and sysadoas in the management of knee osteoarthritis
Source: Aging Clin Exp Res. 2025 Nov 6;37(1):317. doi: 10.1007/s40520-025-03221-2 (PMC12592241; doi:10.1007/s40520-025-03221-2)
Supplement: Supplementary file 3 — Supplementary Material 3 [file 40520_2025_3221_MOESM3_ESM.docx]

| **Author/Year** | **Type of Study** | **Disease/Where** | **Sysadoa** | | **NSAID** | | **n patients** | **Endpoint 1** | **Endpoint 2** | **Follow up** | **Safety** | **Observations** |
| --- | --- | --- | --- | --- | --- | --- | --- | --- | --- | --- | --- | --- |
|  |  |  | **Active ingredients** | **Dosage** | **Drug** | **Dosage** |  |  |  |  |  |  |
| **1** Reginster 2021 | Review | OA (Knee, Hip, Hand) | hpCS | 400 mg/800 mg/1200 mg  (1200mg day) |  |  | around 6000 | This article aims to review the evidence for the role of highly purified (hp) CS (Condrosulf®, IBSA) in the treatment of OA |  | From 3 months to 2 years | The incidence and severity of hpCS-related adverse events (AEs) are low and similar to those of the placebo also at the dosage of 1200 mg/day Highly purified CS proved to be safe and effective in hip, knee and hand OA, acting on signs, symptoms and structural changes. The use of hpCS reduced the use of NSAIDs and their side effects |  |
| **2** Honvo 2019 | Systematic Review and Meta‑Analysis | OA | Various | Various | Diacerein |  |  | aimed to re-assess the safety of various SYSADOAs in a comprehensive meta-analysis of randomized placebo controlled trials, using, as much as possible, data from full safety reports |  | In most of the studies, treatment durations varied between 12 and 26 weeks, with the shortest being 4 weeks and the longest 156 weeks | GS and CS can be considered safe treatments for patients with OA,  Significantly more gastrointestinal disorders were reported with diacerein than with placebo | The safety profile for coadministration of diacerein and oral NSAIDs requires further investigation |
| **3** Simental Mendia 2018 | systematic review and meta-analysis of randomized placebo-controlled trials | symptomatic knee osteoarthritis | CS  GH  placebo | (from 400 to 1200 mg/day)  (1200 mg day) |  |  | 6120 | investigate the efficacy of glucosamine and chondroitin sulfate on knee OA symptoms |  | From 1 month to 2 years | there is no additional effect using both therapeutic agents in combination for the management of symptomatic knee OA | this meta-analysis suggests that supple mentation with glucosamine or chondroitin sulfate reduce pain in knee OA patients. Apparently, there is no addi tional effect when the two oral supplements are coadmin istered. our study exhibits some limitations: first, outcomes were based only in VAS and WOMAC indexes; second, treatment duration in most of the included studies was ≤ 6 months (22 clinical trials); therefore, the long-term effect of glucosamine and chon droitin sulfate cannot be assured; third, the sample size was small in some of the selected trials, however, the pooled sample size included 6120 participants |
| **4** Bruyere 2018 | Prospective, randomized, doublu-blind, double-dummy, placebo controlled trial | Knee OA | CS | 800 mg | Celecoxib  Placebo | 200 mg | 604 | One endpoint was the patient’s estimate of pain on a 100mm Visual Analog Scale (VAS) with the other co-primary endpoint being the Lequesne Index (LI), a composite index which integrates pain and function | Secondary endpoints included the proportion of patients reaching the Minimally Clinically Important Improvement (MCII) and the Patient Acceptable Symptoms State (PASS), two outcomes reflecting the self-perception, by the patients, of the magnitude of the improvement observed in their symptoms | 6 months | All medications showed very good safety profile throughout the study | This study showed that CS is superior to placebo and similar to celecoxib in reducing pain and improving function in Kellgren 1 2 Lawrence grade 1-3 patients supporting the role of pharmaceutical-grade CS as a potential first-line treatment for the management of patients with mild to moderate knee OA |
| **5** Morita 2017 | one-year, randomized, double blind, dose-comparison study | Knee OA | Low dose group CS  High dose group CS | 260/mg day  1560/mg day |  |  | 73 | The aim of the present study was to investigate the pos sible differences in efficacy between two doses of CS in  Japanese patients with radiographically confirmed knee OA  that is symptomatic |  | One year | Many clinical trials have confirmed the excellent safety  profile and good tolerability of CS.31,43,61) High-dose CS  therapy was tolerable in the present study, and there was no increased drop-off rate compared to low-dose therapy. Also consistent with previous studies, we showed that there were no serious adverse effects attributable to CS and that tolerability  was good | we demonstrated oral treatment with sodium CS is tolerable, and that a dose of 1560 mg/d is more effective  than 260 mg/d for pain relief in patients with knee OA  Small size of parecipants |
| **6** Roman Blas 2016 | randomized, double-blind, placebo-controlled clinical trial | Knee OA | CS  GS  Placebo | 1200 mg  1500 mg |  |  | 164 | To assess the efficacy and safety of chondroitin sulfate (CS) plus glucosamine sulfate (GS) compared to placebo in patients with symptomatic knee osteoarthritis (KOA) |  | Daily dose for 6 months | Regarding safety, 97% (N=159) of the patients included in the ITT population received, at least, one dose of the study treatment. Of these, a small number of dropouts related to adverse events was reported in both groups. The incidence was higher in the CS+GS group, and mainly consisted of abdominal complaints such as diarrhoea, upper abdominal pain, and constipation. There was, however, a trend toward fewer withdrawals associated to other reasons in the CS+GS group | CS+GS failed to demonstrate superiority over placebo in reducing pain and function impairment in patients with symptomatic KOA at 6 months |
| **7** Singh 2015 | Review of 43 randomized controlled trials | majority of trials were in knee OA, with few in hip and hand OA | CS | 800 mg/day |  |  | 4,962 participants treated with chondroitin and 4,148 participants given placebo or another control were included | To evaluate the benefit and harm of oral chondroitin for treating osteoarthritis compared with placebo or a comparator oral medication including, but not limited to, nonsteroidal anti-inflammatory drugs (NSAIDs), analgesics, opioids, and glucosamine or other "herbal" medications |  | Trial duration varied from 1 month to 3 years | Chondroitin had a lower risk of serious adverse events compared with control | chondroitin (alone or in combination with glucosamine) was better than placebo in improving pain in participants with osteoarthritis in short-term studies |
| **8** Tsuji 2015 | randomized, double blind, placebo-controlled trial | Knee pain | N-acetyl glucosamine  CS | 100 mg/day  180 mg/day |  |  | 50 | To investigate the effects of 24 week oral N-acetyl glucosamine and chondroitin sulfate supplementation on knee pain, self-reported knee function, physical activity, and physical performance | The secondary outcomes were physical activity and physical performance | 12 weeks | No adverse effect related to the study protocol was observed in the study. The abovementioned Cochrane review [5] investigated toxicity of glucosamine in the pharmacological management of knee OA by collaborating 16 studies and reported no adverse event specific to glu cosamine. We also confirmed the sufficient low toxicity and high safety of 24-week consumption of N-acetyl glu cosamine and chondroitin sulfate in Japanese people | These results suggest that consumption of N acetyl glucosamine and chondroitin sulfate for 12 weeks or longer has a positive effect on self-reported knee function and household physical activity in middle-aged and older Japanese adults with knee pain and/or stiffness |
| **9** Rovati 2016 | PEGASus  Cohort study | Knee OA | Glucosamine sulfate  Glucosamine hydrochloride two tablets, each corresponding to 625 mg (total 1250 mg) glucosamine, once daily Chondroitin sulfate 400 mg capsules or sachets, three times daily Diacerein 50 mg capsules, twice daily ASU 300mg capsules, once daily | 1500 mg/day |  |  | 6000 | The use of Symptomatic Slow-Acting Drugs in Osteoarthritis (SYSADOAs) may be expected to decrease the use of concomitant medications for rescue analgesia, including non-steroidal anti-inflammatory drugs (NSAIDs). The Pharmaco-Epidemiology of GonArthroSis (PEGASus) study was designed to assess this possibility |  | Follow-up visits were as per routine medical practice in the 12 months following enrollment, with telephone interviews after 1 month andat4-month intervals thereafter up to 24 months | There is in fact general concern regarding a possible overuse, especially of NSAIDs, given their poor safety profile on gastrointestinal, cardiovascular, renal, and other systems. | Crystalline glucosamine sulfate was the only SYSADOA that decreased the use of NSAIDs in this pharmaco-epidemiology study in patients with knee OA |
|  |  |  |  |  |  |  |  |  |  |  |  |  |
|  |  |  |  |  |  |  |  |  |  |  |  |  |
|  |  |  |  |  |  |  |  |  |  |  |  |  |
|  |  |  |  |  |  |  |  |  |  |  |  |  |
|  |  |  |  |  |  |  |  |  |  |  |  |  |
|  |  |  |  |  |  |  |  |  |  |  |  |  |
|  |  |  |  |  |  |  |  |  |  |  |  |  |
|  |  |  |  |  |  |  |  |  |  |  |  |  |
|  |  |  |  |  |  |  |  |  |  |  |  |  |
|  |  |  |  |  |  |  |  |  |  |  |  |  |
|  |  |  |  |  |  |  |  |  |  |  |  |  |
|  |  |  |  |  |  |  |  |  |  |  |  |  |
|  |  |  |  |  |  |  |  |  |  |  |  |  |
|  |  |  |  |  |  |  |  |  |  |  |  |  |
|  |  |  |  |  |  |  |  |  |  |  |  |  |
|  |  |  |  |  |  |  |  |  |  |  |  |  |
|  |  |  |  |  |  |  |  |  |  |  |  |  |
