# Supplementary material for: Low dose NSAIDs and sysadoas in the management of knee osteoarthritis
Source: Aging Clin Exp Res. 2025 Nov 6;37(1):317. doi: 10.1007/s40520-025-03221-2 (PMC12592241; doi:10.1007/s40520-025-03221-2)
Supplement: Supplementary file 4 — Supplementary Material 4 [file 40520_2025_3221_MOESM4_ESM.docx]

| **Author/Year** | **Type of Study** | **Disease/Where** | **Sysadoa** | | **NSAID** | | **n patients** | **Endpoint 1** | **Endpoint 2** | **Follow up** | **Safety** | **Observations** |
| --- | --- | --- | --- | --- | --- | --- | --- | --- | --- | --- | --- | --- |
|  |  |  | **Active ingredients** | **Dosage** | **Drug** | **Dosage** |  |  |  |  |  |  |
| **1** De Campos 2021 | systematic review with network meta-analysis (12 RCT) | Osteoarthritis |  |  | celecoxib 200 mg (C200), diclofenac 150 mg (D150) and 100 mg (D100), and ultramicronised diclofenac 105 mg (UD105) and 70 mg (UD70) and placebo  Ultramicronised Diclofenac 35 mg for 2-3 times for day | | 4767 | This systematic re view with network meta-analysis was performed to compare the effectiveness of oral anti-inflam matory drugs used in Brazil for osteoarthritis |  | Various but not for low doses | Treatments was well tollereted in all cases | Ultramicronised diclofenac demonstrated superior efficacy compared to other conventional anti-inflammatory drugs and placebo in relieving osteoarthritis pain |
| **2** Cooper 2019 | Narrative review | OA |  |  | Diclofenac | 150 |  | In this narrative literature review, we have identified data on the safety of traditional nsNSAIDs (naproxen, ibuprofen, diclofenac) published since the Cochrane review of 2011, to identify current understanding on the relative risk:benefit of the use of nsNSAIDs to manage pain in OA |  |  | All NSAIDs have the potential for GI and CV toxicity through their action on the COX-1 and COX-2 enzymes. If nsN SAIDs are taken with a gastroprotective proton pump inhibi tor, the upper GI toxicity is attenuated and similar to that found with a COX-2-specific NSAID. There is an increased risk of acute MI with all NSAIDs, which may occur within 7 days of use. The risk of incident HF is elevated with all NSAIDs. An increased risk of hemorrhagic stroke appears to be limited to the nsNSAIDs with the highest COX-2 selec tivity, diclofenac and meloxicam. All nsNSAIDs are associ ated with an increased risk of AKI. While opioid analgesics are associated with an increased risk of falls and fractures, NSAIDs are associated to a lesser extent. The excess mor tality observed with OA may be attributable, in part, to treatment algorithms including NSAIDs, paracetamol, and possibly COX-2 inhibitors | multiple strategies to control symptoms in OA should be considered on an individual patient basis |
| **3** Pelletier 2016 | Review | OA |  |  | Various |  |  | Efficacy and safety of oral NSAIDs and analgesics in the management of osteoarthritis |  |  | The NSAIDs are non-homogeneous as a drug class, and there are vast differences between individual drugs in terms of adverse event (AE) risk for gastrointestinal (GI) and cardiovascular (CV) complications. Thus, the benefit-risk balance of individual NSAIDs is mainly driven by their GI and CV safety profile. The US Food and Drug Administration (FDA) has issued a “black box” safety warning for the entire oral NSAID drug class highlighting the potential for increased risk of CV events and GI bleeding associated with their use | A recent short-term trial of low dose celecoxib (200 mg/day) found that continuous treatment with celecoxib over 22 weeks was significantly more effective than intermittent use in preventing OA flares of the hip and knee without an increase in overall AEs including GI disorders and hypertension. However, the select population included in the trial had low CV and GI risks and, therefore, is not representative of the general OA patient population |
| **4** Ueberall 2016 | Review of six randomized controlled trials | OA of Knee |  |  | Diclofenac | Daily dose from 100 to 150 mg | 774 | To compare efficacy, safety, and tolerability of an oral enzyme combination (OEC) containing proteolytic enzymes and bioflavonoid vs diclofenac (DIC), a nonselective nonsteroidal anti-inflammatory drug in the treatment of osteoarthritis of the knee |  | Treatments were given daily for a planned duration of 3 weeks in three studies, 6 weeks in two, and 12 weeks in one study | This overall superior risk–benefit profile qualifies OEC not only as an alternative OA treatment to DIC and other NSAIDs in high-risk patients but also as a safe and efficacious option for the daily management of OA-related joint pain | When compared with DIC, OEC showed comparable efficacy and a superior tol erability/safety profile associated with a significantly lower risk of treatment-emergent adverse events, related study discontinuations, and changes in laboratory parameters |
|  |  |  |  |  |  |  |  |  |  |  |  |  |
|  |  |  |  |  |  |  |  |  |  |  |  |  |
|  |  |  |  |  |  |  |  |  |  |  |  |  |
|  |  |  |  |  |  |  |  |  |  |  |  |  |
|  |  |  |  |  |  |  |  |  |  |  |  |  |
|  |  |  |  |  |  |  |  |  |  |  |  |  |
|  |  |  |  |  |  |  |  |  |  |  |  |  |
|  |  |  |  |  |  |  |  |  |  |  |  |  |
|  |  |  |  |  |  |  |  |  |  |  |  |  |
|  |  |  |  |  |  |  |  |  |  |  |  |  |
|  |  |  |  |  |  |  |  |  |  |  |  |  |
|  |  |  |  |  |  |  |  |  |  |  |  |  |
|  |  |  |  |  |  |  |  |  |  |  |  |  |
|  |  |  |  |  |  |  |  |  |  |  |  |  |
|  |  |  |  |  |  |  |  |  |  |  |  |  |
|  |  |  |  |  |  |  |  |  |  |  |  |  |
|  |  |  |  |  |  |  |  |  |  |  |  |  |
|  |  |  |  |  |  |  |  |  |  |  |  |  |
|  |  |  |  |  |  |  |  |  |  |  |  |  |
|  |  |  |  |  |  |  |  |  |  |  |  |  |
|  |  |  |  |  |  |  |  |  |  |  |  |  |
